# Recent Advances in Transcriptome Analysis Within the Realm of Low Arsenic Rice Breeding

**DOI:** 10.3390/plants14040606

**Published:** 2025-02-17

**Authors:** Guanrong Huang, Guoping Yu, Huijuan Li, Haipeng Yu, Zengying Huang, Lu Tang, Pengfei Yang, Zhengzheng Zhong, Guocheng Hu, Peng Zhang, Hanhua Tong

**Affiliations:** 1 State Key Laboratory of Rice Biology, China National Rice Research Institute, Hangzhou 310006, China; 13417992759@163.com (G.H.); lihuijuan0812@163.com (H.L.); YuHaipengCNRRI@163.com (H.Y.); tenzyiii@163.com (Z.H.); 18570360851@163.com (L.T.); ypf19990703@163.com (P.Y.); zhongzhengzheng@caas.cn (Z.Z.); huguocheng@caas.cn (G.H.); 2 National Nanfan Research Institute, Chinese Academy of Agricultural Sciences, Sanya 572024, China; yuguoping@caas.cn

**Keywords:** arsenic toxicity, transcriptome analysis, low-arsenic breeding, rice (*Oryza sativa* L.), plant growth

## Abstract

Arsenic (As), a toxic element, is widely distributed in soil and irrigation water. Rice (*Oryza sativa* L.), the staple food in Southern China, exhibits a greater propensity for As uptake compared to other crops. Arsenic pollution in paddy fields not only impairs rice growth but also poses a serious threat to food security and human health. Nevertheless, the molecular mechanism underlying the response to As toxicity has not been completely revealed until now. Transcriptome analysis represents a powerful tool for revealing the mechanisms conferring phenotype formation and is widely employed in crop breeding. Consequently, this review focuses on the recent advances in transcriptome analysis within the realm of low As breeding in rice. It particularly highlights the applications of transcriptome analysis in identifying genes responsive to As toxicity, revealing gene interaction regulatory modules and analyzing secondary metabolite biosynthesis pathways. Furthermore, the molecular mechanisms underlying rice As tolerance are updated, and the recent outcomes in low As breeding are summarized. Finally, the challenges associated with applying transcriptome analysis to low-As breeding are deliberated upon, and future research directions are envisioned, with the aim of providing references to expedite high-yield and low-arsenic breeding in rice.

## 1. Introduction

Arsenic (As), a highly toxic metal, predominantly occurs in paddy fields as two main inorganic forms, i.e., arsenite [As(III)] and arsenate [As(V)], along with relatively common organic forms like monomethyl arsenic acid (MMA), dimethyl arsenic acid (DMA) and trimethyl arsine acid (TMA) [1,2]. These As forms can be interconverted in accordance with redox potentials [1,2]. Among them, As(III) exhibits the highest toxicity, followed by As(V) and then organic As [3]. As the top of the food chain, humans mainly ingest As by consuming crops cultivated in contaminated soil and drinking contaminated water [4,5,6]. Long-term exposure to such As sources readily leads to chronic poisoning and even cancer. Unfortunately, As pollution is prevalent in numerous countries globally (including China) as a result of geotectonic movement and human activities [7]. In some countries, the As concentrations in water sources seriously exceed the WHO standard limit (10 μg/L), posing a threat to human health [3,4]. For example, in 61 out of 64 districts in Bangladesh, the As levels in drinking water exceeded the standard limit, affecting more than 85 million people (arsenicosis occurred mostly among the poor) and causing a series of social and economic problems [8,9]. Even more alarmingly, it has been reported that the average As content of groundwater in Kandal Province, Cambodia, was as high as 178 μg/L [10]; China is one of the countries heavily affected by As-contaminated groundwater, according to the investigation, about 19.6 million people have long-term exposure to As-contaminated groundwater, and about 10,000 people have been diagnosed with As poisoning [11]. Thus, As pollution has emerged as a severe global issue [12].

Cereal crops such as rice (*Oryza sativa* L.), wheat (*Triticum aestivum* L.) and barley (*Hordeum vulgare* L.) display a notably higher efficiency in As accumulation within grains and shoots compared to other crops, with the former one exhibiting the most proficient As uptake [2,13,14]. Rice growth is significantly weakened and may even die when exposed to As toxicity, which is mainly caused by the disruption of photosynthesis [15,16]. In order to sustain basic viability, stressed plants will actively make a series of avoidance and tolerance responses, such as promoting the As ion storage in the vacuole and their efflux outward [17]. Measures can be taken to prevent/mitigate As toxicity in rice production. Firstly, without affecting rice growth, agronomic measures such as flooding irrigation, alternating wet and dry irrigation and the “phosphorus-control and silicon-increase” fertilization regime can be employed to impede the ingress of As ions into the root cells or promote their fixation in the soil [18,19,20,21,22,23]. Secondly, exogenously applied soil amendments (e.g., activated carbon [24], modified biochar [25] and iron oxides [26]) adsorb and/or precipitate As ions in the soil, thereby diminishing their bioavailability. Thirdly, As bioavailability can be reduced by appropriately increasing soil pH (through the application of alkaline substances such as lime) [27] and/or redox potential (applying oxidizing agents such as potassium permanganate and calcium peroxide, etc.) [28]. Fourthly, As-contaminated soil can be efficaciously remediated via cultivating hyperaccumulator plants such as *Pteris vittata* [29,30], *Pteris cretica* [31], *Pteris longifolia* [32], etc. Fifthly, the microbial remediation of contaminated soils mitigates rice As toxicity through biosorption, fixation, redox reactions, methylation and modulation of the inter-root environment [33,34,35,36,37]. Comparatively, exogenously applied soil amendments (the application of goethite-modified biochar reduced the As content in rice grain by 77% [38]), microbial remediation (the application of iron-oxidizing bacteria reduced the As content in rice grain by 62.5% [39]) and agronomic measures (under continuous aerobic treatment, the inorganic and organic As contents in rice grains were reduced by 84% and 81%, respectively [18,23]) are more suitable and convenient for application in agricultural production. Phytoremediation and pH adjustment are also effective measures, but the former requires a long time and a specific growing environment, and the latter negatively affects soil microbial activity and the efficacy of other nutrients, making them difficult to apply in practical agricultural production [40]. In any case, it is essential for crops to possess inherent As tolerance. Therefore, there is an urgent need to breed high-yield rice varieties with low As content.

With the development of science and technology and the reduction of sequencing costs, integrating forward genetics studies and transcriptome analysis have been widely used to identify causal genes controlling plant phenotypes, analyze their corresponding genetic regulatory mechanisms and develop molecular markers. The main tasks of transcriptome analysis include classifying all transcripts, ascertaining the transcriptional structure of each gene and quantifying the expression fluctuations of each transcript [41]. Transcriptome analysis has become a powerful tool for characterizing agronomic traits and accelerating the breeding process, owing to its capacity to reveal gene expression differences in a rapid, convenient, comprehensive and systematic manner.

Arsenic toxicity imperils not only human health but also poses a grave menace to food security. Comprehensively revealing the molecular mechanisms underlying As toxicity is the key to low As breeding in rice. However, in contrast to other traits (e.g., yield traits, plant type traits), As uptake, transport and metabolism have not been widely studied in rice (Table 1 and Table 2), and few genetic resources are available (for details, refer to Section 2.3); moreover, forward genetics studies entail a protracted duration and high costs to identify target genes and corresponding regulatory modules. Hence, there is an exigency to integrate omics approaches, with transcriptome analysis being a prime example. This review highlights transcriptome analysis and its applications in identifying As stress-responsive genes, revealing gene regulatory modules and analyzing the biosynthetic pathways of differentially expressed genes (DEGs) while summarizing its challenges and discussing the gaps that require further research. It is expected to provide references for low As breeding in rice.

## 2. The Application of Transcriptome Analysis in Low-As Rice Breeding

Arsenic, a toxic metallic element, provides no nutrients to plants; rather, it disrupts plant metabolism and growth. Presently, the impacts, mitigation strategies and metabolic molecular mechanisms of rice As toxicity have been widely reviewed [2,40,89,90]. Thus, in this section, we focus on reviewing the recent advances in the application of transcriptome analysis for low As rice breeding. Additionally, we briefly refine the biosynthetic pathways in response to As toxicity in rice; finally, we summarize the recently reported discoveries in low-As breeding.

### 2.1. Secondary Metabolite Biosynthesis Pathways for as Tolerance in Rice

Comparing the transcriptome data of various rice tissues before and after treatment with different As concentrations, thousands of As-responsive DEGs were found. These DEGs are involved in various pathways (including photosynthesis, transcription factors, defense mechanisms, redox homeostasis, signaling, transporter proteins, metallothioneins, heat shock proteins, protein kinase activity, phosphorylation and detoxification, etc.), as well as multiple metabolic pathways (including hormones, lipids, amino acids, sulfate, phosphorus, nucleotides, terpenes and polyketides, etc.) [91,92,93,94,95,96,97,98]. It suggests that the molecular mechanisms conferring rice plants’ response to As toxicity are extremely complex. Briefly, the As tolerance of rice plants is maintained by many key DEGs that form a complex and balanced regulatory network. In an As-containing environment, if the transcriptional activity of a key DEG, such as *OsHAG1* [73] (for details, refer to Section 2.2.1), is lost, this balance will be disrupted, simultaneously affecting other key DEGs and resulting in the reduction or even loss of an individual’s As tolerance. Likewise, by integrating transcriptome sequencing data and available gene-function information, we can predict the phenotypic changes that may occur in organisms under specific gene-environment interactions.

Transcriptome analysis generates copious amounts of data and numerous DEGs involved in the stress response. Therefore, the representative secondary metabolite biosynthesis pathways and responsive genes can not be identified, nor can the change beyond the RNA dimension in the stressed plant be discovered. Integrating other omics approaches is one of the most vital ways to solve this problem. As an example, Ma et al. [99] integrated transcriptomic and metabolomic technologies and found that differences were significantly co-enriched in the pathways of “photosynthetic biogenic carbon sequestration”, “phenylalanine, tyrosine, and tryptophan biosynthesis”, “ABC transporter protein” and “metabolism of α-linolenic acid, linoleic acid, purine, alanine, aspartic acid and glutamate” by KEGG co-enrichment analysis. Likewise, Awasthi et al. [100] joined transcriptomic and proteomic technologies and revealed that the common differences were mostly enriched in “photosynthesis”, “reactive oxygen species and defense”, “cellular signaling”, “energy metabolism”, “amino acid metabolism”, “translocation” and “protein synthesis” pathways. Evidently, As toxicity primarily disrupts photosynthesis in rice plants.

Plants are organic entities wherein the elements within the tissues are interdependent, and they adapt to the outside environment by regulating the elemental balance under stress. A study combined transcriptomics and ionomics technologies and discovered that As-stressed rice plants maintained the dynamic equilibrium of phosphorus in shoots by up-regulating the expression of genes involved in “protein kinase activity”, “phosphorus metabolism” and “phosphorylation” pathways. Concurrently, As toxicity inhibited the translocation of zinc and calcium from the roots to shoots, triggering the up-regulation of the expression of zinc- and calcium-binding genes, which maintained the mineral nutrient homeostasis during the plant’s essential metabolic processes [101]. Another study further demonstrated that ionome data and transcriptome data were tightly associated and that the number of significant correlations forming the ionome network within rice tissues diminished under As(III) toxicity, predominantly attributable to a dramatic increase in the expression of genes controlling ion transporter proteins and ion binding proteins [102]. These results indicate that the concentration of each ion within the plant changes drastically when rice is exposed to As toxicity, and a novel balance is subsequently established.

Taken together, the diminished photosynthetic capacity of stressed plants leads to feeble growth potential when rice plants are exposed to an As environment; only basic survival is maintained via the modulation of mineral nutrient homeostasis and amino acid metabolism in vivo. The above information provides important insights to further reveal the precise molecular mechanisms underlying rice’s response to As toxicity.

### 2.2. Identification of Genes Related to as Tolerance in Rice

#### 2.2.1. Candidate Genes Solely Identified by Transcriptome Analysis

**Figure 1:**
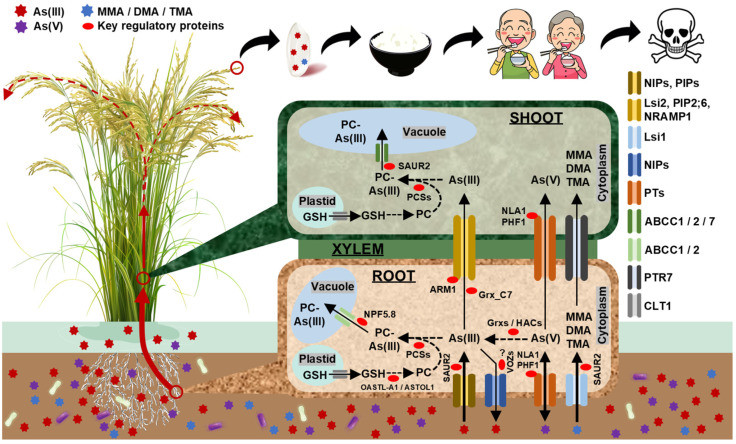
Molecular mechanisms of As uptake, transport and metabolism in rice. NIPs consist of proteins encoded by genes *NIP1;1*, *NIP3;1*, *NIP3;2*, *NIP3;3*, *NIP2;1* (*Lsi1*) and *NIP2;2* (*Lsi2*). PIPs consist of proteins encoded by genes *PIP1;2*, *PIP1;3*, *PIP2;4*, *PIP2;6* and *PIP2;7*. PTs consist of proteins encoded by genes *PT1*, *PT2*, *PT4* and *PT8*. ABCs consist of proteins encoded by genes *ABCC1*, *ABCC2* and *ABCC7*. PCSs consist of proteins encoded by genes PCS1 and PCS2. Grxs consist of proteins encoded by genes *Grx_C2.1* and *Grx_C7*. HACs consist of proteins encoded by genes *HAC1;1*, *HAC1;2* and *HAC4*. VOZs consist of proteins encoded by genes *VOZ1* and *VOZ2*. For details, please see Table 2. As(V), As(III), MMA, DMA, TMA, PC, GSH, NIPs, PIPs, PTs, ABCs, PCSs, Grxs, HACs and VOZs are abbreviations for arsenite, arsenate, monomethyl arsenic acid, dimethyl arsenic acid, trimethyl arsenic acid, phytochelatin, glutathione, nodulin 26-like intrinsic proteins, plasma membrane intrinsic proteins, phosphate transporters, C-type ATP-binding cassette transporters, phytochelatin synthase, glutaredoxins, high As concentrations and transcription factor VOZs, respectively.

Transcriptome analysis holds significant advantages in identifying candidate genes. As early as 2009, Chakrabarty et al. [92] identified 72 and 27 candidate genes in response to As(V) and As(III), respectively, using transcriptome analysis integrated with gene annotation and available literature descriptions, including the subsequently cloned *OsGrx_C7* [71], *OsGrx_C2.1* [71] and *OsWRKY28* [78] (Figure 1, Table 2). Among them, the former two are genes encoding glutaredoxins (Grxs), which possess arsenate reductase activity and catalyze the conversion of intracellular As(V) to As(III). Overexpression of these two genes reduces As content in rice grains [71], while *OsWRKY28* (a gene encoding WRKY transcription factor) negatively regulates the As content of rice shoots (Table 2) [78]. Besides inhibiting the conversion of As(V) to As(III), *OsGrx_C7* also indirectly negatively mediates the aquaporins, i.e., silicon efflux transporter (*OsLsi2*), aquaporin PIP2-6 (*OsPIP2;6*) and metal transporter Nramp1-like (*OsNRAMP1*), thereby inhibiting the translocation of As(III) from root to aboveground parts (Figure 1) [72]. Recently, Yang et al. [73] confirmed that the down-regulation of *OsABCC1* (a key gene encoding C-type ATP-binding cassette transporter) in *hag1* (a rice mutant) resulted in increased As content in rice grains through comparative transcriptome analysis of *hag1* and wild-type material (WT); furthermore, the mutation of *OsHAG1* gene induced changes in the transcript abundance of other functional genes, including those encoding C-type ATP binding cassettes (ABCs), nodulin 26-like intrinsic proteins (NIPs), plasma membrane intrinsic proteins (PIPs), arsenate reductases (i.e., high As concentrations, HACs) and phosphate transporters (PTs) (Figure 1, Table 2).

**Figure 2:**
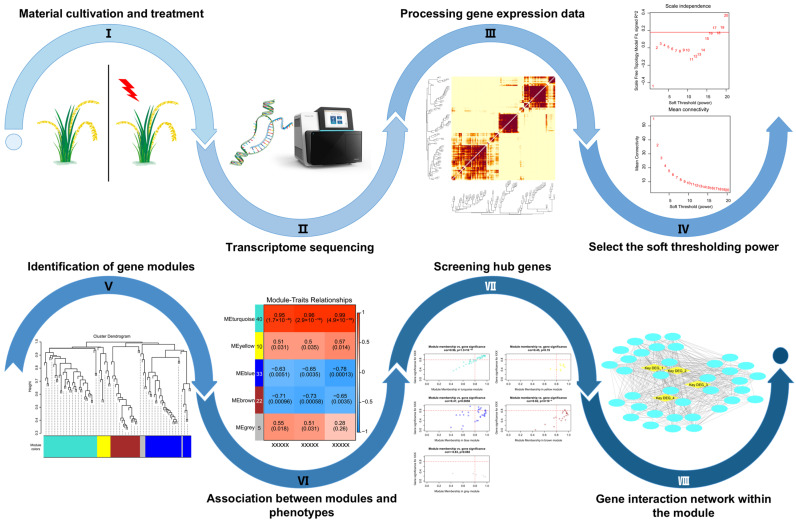
The flow diagram of WGCNA.

Transcriptome analysis yields massive amounts of data and requires reliable analytical and statistical methods for prediction; without such methods, it is difficult to identify the main-effect genes. For example, Sehar et al. [95] identified dozens of genes within key biosynthesis pathways “hormones”, “efflux proteins” and “detoxification”; in addition, 3860 genes with unknown functions were found. An excessive number of candidate genes were obtained, rendering it challenging to conduct functional validation and apply them in breeding. Weighted gene co-expression network analysis (WGCNA), a systematic biological method, is employed to characterize gene association patterns among samples, which can be utilized to identify highly synergistic gene sets and detect candidate genes based on gene set internal-linkage and gene set-phenotype associations (Figure 2). By way of a representative example, Xu et al. [96] compared the transcriptome data of plants prior to and subsequent to stress (simultaneous treatment with microplastic and As). Using WGCNA, they classified DEGs into distinct modules and identified several hub genes therein, including genes encoding photosynthesizing enzymes, protein kinases and transcription factors; these genes represent potential targets for low-As breeding in rice. Similarly, Lim et al. [84] investigated the functional interactions of all candidate genes through comparative transcriptome analysis between *ATT1* mutant and WT using WGCNA; eventually, they identified *OsNPF5.8* (a gene encoding nitrate transporter 1/peptide transporter family 5.8) as the causal gene. *ATT1* mutant enhances plant As tolerance by increasing cell number, enlarging cell size and facilitating the translocation of phytochelatins (PC)-As(III) complexes into the vacuole (Figure 1, Table 2) [84].

A few strongly and stably responsive DEGs can be identified from a vast quantity of genes by integrating three statistical methods, i.e., linear models for microarray data (LIMMA) [103], significance analysis of microarrays (SAM) [104,105] and *T*-test, when transcriptome analysis is performed using cDNA microarray technology. This integration greatly reduces the number of minor-effect and/or false-positive genes. For instance, Brinke et al. [106] employed this strategy to extract 53 DEGs from over 16,000 DEGs, among which three DEGs (i.e., *UDPGT*, *pollenless3* and *OsABI5*) were screened as candidate biomarker genes.

#### 2.2.2. Candidate Gene Identified by Transcriptomic Analysis Combined with Other Omics Approaches

Currently, multi-omics analysis serves as an indispensable aid in revealing the genetic mechanism conferring crop phenotypes. The combination of transcriptome analysis with other omics approaches further narrows down the range of candidate DEGs and their secondary metabolite biosynthesis pathways, thereby reflecting the differences between comparator groups more comprehensively and accurately. To give a visual example, Ma et al. [99] identified thousands of DEGs through comparative transcriptome analysis; subsequently, upon further integration with metabolome analysis, 42 candidate DEGs were found within the co-enriched KEGG pathway; among these candidates, the majority (down-regulated) were concentrated in the “photosynthetic biogenic carbon sequestration” pathway, while some up-regulated DEGs were clustered in the “phenylalanine, tyrosine and tryptophan biosynthesis” pathway. This approach significantly mitigates the interference from redundant data and facilitates the screening of key DEGs.

Quantitative trait locus (QTL) and/or genome-wide association studies (GWAS) based on whole-genome resequencing combined with transcriptome analysis and/or other omics approaches have been widely used in gene mining. Shortly ago, Lee et al. [46] conducted whole-genome resequencing and transcriptome analysis on 15-day-old grains after heading from 273 materials, and 12 candidate genes were identified in 23 co-expression networks of reported functional genes via GWAS and transcriptome-wide association studies (TWAS); furthermore, the *cis*-eQTL of AIR2 (arsenic-induced RING finger protein), an important protein contributed to the divergence in As content between *indica* and *japonica* rice grains, was isolated using TWAS. In fact, there are comparatively few reports related to As QTLs in rice, and in the majority of these cases, candidate genes were not further mined (Table 1). Although some important functional genes have been found (Figure 1, Table 2), only a limited number of target genes are applied in breeding (for details, refer to Section 2.3), together with the complex molecular mechanism of As tolerance; therefore, subsequent studies ought to center on “the integration of transcriptome analysis and main-effect QTLs” as one of the focuses.

#### 2.2.3. Candidate Interacting Genes Identified by Transcriptome Analysis

A group of candidate interacting genes can be effectively screened by comparative transcriptome analysis of WT and knockout and/or overexpression mutants. To give a successful example, Wang et al. [78] found, through transcriptome analysis of WT and *oswrky28* lines, that the knockout of *OsWRKY28* led to significant differences in the expression of genes controlling the biosynthesis of the phytohormone, particularly jasmonic acid. Subsequently, they identified five up-regulated genes involved in the jasmonic acid biosynthesis pathway (namely *Os02g0106100*, *Os06g0569500*, *Os06g0728700*, *Os02g0194700* and *Os12g0559200*), which potentially interact with *OsWRKY28*. Likewise, Wen et al. [86] discovered that the knockout of *OsVOZ1* and/or *OsVOZ2* (genes encoding transcription factor VOZs) reduced the As content in rice grains (Table 2) and hypothesized that *OsVOZs* regulate As transport through post-translational modification of As transporter proteins because no As transport-related DEGs were found (that is, OsVOZs proteins do not directly interact with As transporter proteins) (Figure 1). Actually, this strategy does not invariably yield success. Its efficacy depends more on mastering enough information about the target gene. For instance, one needs to accurately judge the biosynthetic pathway in which the target gene is involved based on the phenotypic and/or physiological differences between WT and mutants and then screens for candidate interacting genes from DEGs enriched in that pathway.

Certainly, it is necessary to further validate the interactions between the target gene and candidate interacting genes by combining assay yeast single/double hybrid (chromatin) immunoprecipitation, luciferase reporter gene, bimolecular fluorescence complementation, etc. Undoubtedly, performing transcriptome analyses is a simpler, faster and more cost-effective method (at least providing screenable candidate interacting genes) in comparison to assays like yeast two-hybrid sieve libraries.

#### 2.2.4. Other Genes Controlling as Uptake, Transport and Metabolism in Rice

The majority of genes controlling rice As uptake, transport and metabolism have been described by Mawia et al. [2] and Zaidi et al. [89]. Herein, we mainly described the unclassified functional genes (Table 2). *OsNPF5.8* [84], *OsVOZ1* [86], *OsVOZ2* [86] and *OsHAG1* [73] have been described in the previous sections. Apart from *OsHIR1* (a gene encoding hypersensitive-induced reaction protein 1/RING E3 ligase) [81], *OsNLA1*, a novel gene encoding RING-type E3 ubiquitin-protein ligase BAH1-like 2, assumes a crucial role in As uptake and tolerance via negatively regulating the number of PTs to reduce the As content in stressed rice plants (Figure 1) [82]. *SNAC3*, a gene encoding NAC domain-containing protein 75, positively regulates As tolerance and grain yield by modulating the expression of genes controlling related antioxidants, photosynthesis, osmolyte accumulation and stress in rice [88]. *OsNIP3;1*, a gene encoding NOD26-like intrinsic protein 3;1, negatively controls the As content in various tissues (including grains) of rice [59].

Excessive ingestion of elemental As poses a threat to human health. Conversely, selenium is an essential trace element that plays a pivotal role in enhancing human immune function and exerting antioxidant effects to retard aging. The low selenium content in rice grains fails to meet the body’s selenium requirements. Increasing the selenium content in rice grains holds significant implications for improving the selenium nutritional status of our body. Luckily, Sun et al. [68] screened an arsenic-tolerant semi-dominant mutant, *astol1*, from a library of EMS-induced rice mutants, and cloned the causal gene (*OsASTOL1*, a gene encoding cysteine synthase) via forward genetics; the allelic mutation of *OsASTOL1* modulates the uptake of selenium and sulfur and promotes the synthesis of cysteine and PC in rice, thereby achieving multiple effects, including As tolerance in rice plants and reduction in As content, as well as selenium enrichment and sulfur enrichments in rice grains. *OsASTOL1* represents a valuable genetic resource for breeding new low-arsenic and selenium-enriched rice varieties.

In rice production, it is common to reduce plant As intake via bioremediation technologies. Methods like the exogenous application of minerals, organics, nutrients and hormones also prove efficacious in mitigating As toxicity [40]. Nevertheless, these measures fail to remediate As-contaminated soils; instead, they might even increase the mobility and bioavailability of As. Simultaneously harvesting low-As rice grains and remediating contaminated soil is the key to resolving the issue. *OsSAUR2*, a gene encoding auxin-induced protein 10A5, emerges at a propitious moment. Knockout of *OsSAUR2* increased plant root secretions (including homovanillic acid and 3-coumaric acid) and promoted microbial aggregation, which in turn activated As in the contaminated soil; importantly, *OsSAUR2* negatively regulated *Lsi1* (a gene encoding aquaporin NIP2-1-like) and *Lsi2* in roots (i.e., enhancing As uptake in the knockout line) and *OsABCC1* in shoots (i.e., promoting the storage of As in vacuoles in the knockout line) (Figure 1); consequently, the effect of reduced As content in rice grains and increased As content in rice shoots and roots was accomplished [87]. *OsSAUR2* has great potential for simultaneously cultivating low-As rice varieties and remediating contaminated soils.

Other functional genes, which are key genes involved in As uptake, transport and metabolism in rice, are listed in Table 2. They have been well described by Mawia et al. [2] and Zaidi et al. [89]. When rice plants are exposed to an As-contaminated environment, the stressed plants maintain their survival by modifying the expression of these functional genes [73,95,100].

### 2.3. Advances in Low-As Rice Breeding

Germplasm resources constitute the basic materials for breeding. Murugaiyan et al. [107] systematically assessed 46 materials (53 genotypes) with respect to their As tolerance and the As content in rice grains and ultimately screened five materials with high As tolerance and low As content in grains. Similarly, six low-As rice materials were screened from a collection of 313 rice germplasm by Dong et al. [43]. AIR2 is a key protein responsible for divergence in As content between *indica* and *japonica* rice grains (a lower expression of AIR2 correlates with reduced As content) based on which 12 low-As varieties with diminished AIR2 expression were screened from 273 core germplasm by Lee et al. [46].

In comparison with traditional breeding, transgenic breeding can rapidly introduce specific superior genes into recipient plants, breaking species boundaries and achieving more efficient variety improvement with strong relevance and timeliness. In the current era of rapid development, it is imperative for us to master transgenic breeding technology and generate outcomes to safeguard the future, although China adopts a cautious stance towards it, and the public expresses concerns regarding its food safety and ecological implications. To give a well-analyzed example of transgenic breeding, *OsASTOL1* is a beneficial gene that facilitates low As and selenium enrichment in rice grains; however, the excessive synthesis of cysteine leads to feeble plant growth and dramatically reduced yields [68]. Therefore, Xu et al. [108] drove the expression of *astol1* using the promoter of *OsGPX1* (a gene encoding phospholipid hydroperoxide glutathione peroxidase) with a medium expression level to avoid synthesizing excessive cysteine. The *pOsGPX1::astol1* transgenic plants exhibited no significant impact on growth and grain yield and also decreased the As content (16–51%) and increased the selenium content (37–171%), sulfur content (67–81%) and other nutritional elements (including nitrogen, potassium, calcium, iron and copper) content in rice grains.

Genome-modification breeding enables precise, efficient and rapid targeted modification and improvement of the original genes within organisms without introducing exogenous genes. It exhibits enhanced safety compared to transgenic breeding. In 2024, scientists reported several outcomes of genome-modification breeding, implying that it has become one of the mainstream breeding approaches. Herein, we present several well-studied examples of genome-modification breeding. Singh et al. [59] generated an *osnip3;1* knockout line with markedly reduced As content in various tissues using clustered regularly interspaced short palindromic repeats–CRISPR-associated protein 9 (CRISPR-Cas9) technology, identifying *OsNIP3;1* as a prospective target for regulating As content while maintaining unaffected grain yield and plant vigor in rice. Xu et al. [109] edited the C-terminal coding sequence of *Lsi2* via CRISPR-Cas9 technology, and the resultant knockout line showed no significant change in yield and a great reduction in As(III) content in grains (with a 63% decrease in total As); whereas editing *OsLsi1* did not lead to a reduction in As content in grains. Notably, Hu et al. [110] crossed the *osnramp5*-edited line with unaffected agronomic traits (a two-line sterile line with the edited *OsNramp5* controlling cadmium transport in the C815S background) and the *oslsi2*-edited line (a restorer line with the edited *OsLsi2* in the YK17 and C815S backgrounds). Subsequently, they obtained a total of four hybrid combinations [including the novel rice variety TLHR (two-line hybrid rice) with ultra-low grain cadmium and As accumulation] carrying different alleles; these hybrid combinations exhibited significantly reduced As and/or cadmium content compared to CLY17 (a control which generated by crossing YK17 and C815S), while also maintaining a relatively stable yield potential.

Transgenic breeding and genome-modification breeding have evidently provided great convenience for crop improvement. However, due to the public’s concerns about “transgenic” and “genome-modification”, the related transformation results have difficulty entering the Chinese market. Transgenics is an “anti-natural” technology (involving the introduction of exogenous genes), and exogenous genes may translate new proteins, potentially triggering allergic reactions. Moreover, transgenic crops may hybridize with wild relatives via pollen transmission, resulting in genetic pollution that threatens biodiversity. Notably, the long-term cultivation of transgenic crops may cause the evolution of resistance in the target organisms [111]. In contrast, genome modification only modifies the organism’s own genes, making it closer to the “naturalness” of traditional breeding, but there is a risk of off-targeting (accidentally modifying non-targeted genes, resulting in unknown side effects) and a lack of sufficient validation of the long-term food safety of genome-edited crops; furthermore, if the modified genes escape into wild populations, the ecological balance will be severely disrupted [112]. In the future, the development of these two technologies should be coordinated with ethical considerations to accelerate the achievement of a “win–win” situation for sustainable agricultural development.

Presently, China has allocated substantial funds to bolster scientific research and innovation in transgenic technology and genome-modification technology, aiming to enhance China’s core technological competitiveness in this realm and provide technological reserves for agricultural advancement. Not all functional genes are suitable for serving as breeding targets (e.g., *OsLsi1*). Future efforts should focus on validating others, such as *OsPIPs* and *OsNPF5.8* (genes whose mutations increase plant biomass), *SNAC3* (a gene whose mutation increases grain yield) and *OsSAUR2* (a gene whose mutation has the potential to remediate As-contaminated soils) (Table 2).

## 3. Challenges of Transcriptome Analysis Assisting Low-As Breeding in Rice

The accuracy of transcriptome sequencing technology is beset with several limitations. For example, insufficient sequencing depth hampers a comprehensive understanding of gene expression, while uneven coverage might lead to the omission or inaccurate estimation of certain gene expression information. Furthermore, the current transcriptome sequencing technology falls short of perfection when it comes to analyzing complex gene structures and phenomena like alternative splicing, potentially compromising the accurate judgment of related gene functions.

The reproducibility of transcriptome sequencing technology requires further improvement. Except for the differences in samples themselves (e.g., biological noise caused by individual differences, sampling time and freezing method), differences in sequencing technology between platforms can also lead to data errors; furthermore, differences in data analysis, such as the use of different alignment tools, variance analysis software and parameter thresholds for filtering DEGs, may lead to divergent results; the update of the database for enrichment analysis may also cause inconsistent interpretations of the results. Artificial Intelligence (AI) technology is currently developing rapidly and may be able to utilize its capabilities to address data reproducibility issues in the future. For example, AI technology can be used to develop automated data preprocessing tools and software packages that integrate multiple analysis methods, thereby establishing a standardized data analysis process; subsequently, AI technology can be utilized to build a transcriptome data integration platform to collect and standardize transcriptome sequencing data from different units, enabling researchers to query, compare and reanalyze the data.

The molecular mechanisms underlying rice As tolerance are highly complex. Transcriptome analysis yields thousands of DEGs, but the interactions among genes are so complex that it is difficult to analyze them merely by comparing transcriptome data. Moreover, it fails to reveal how post-transcriptional regulatory mechanisms respond to As toxicity, thereby limiting the profound understanding and utilization of genes related to As uptake, transport and metabolism.

Powerful bioinformatics and computational capabilities are requisite for processing and analyzing the data. Transcriptome analysis yields massive amounts of data. Effectively integrating, analyzing and mining these data to extract key information thereby poses a tremendous challenge.

It demands profound biological knowledge and rich research experience. Even when the crucial information (e.g., DEGs and their associated pathways) is retrieved from the massive data, it remains challenging to precisely interpret their biological significance and establish connections to the As toxicity response mechanisms in rice.

## 4. Future Perspectives and Conclusions

Combining transcriptome analysis with forward genetics studies, we can further mine functional genes and their corresponding regulatory modules. The functional genes identified to date merely represent the tip of the iceberg (Table 2). In the future, we ought to systematically mine novel genes and their corresponding regulatory modules in response to As toxicity via more in-depth transcriptome analysis, combined with methods such as QTL mapping, GWAS, gene knockdown, gene overexpression, etc.

Achieving a comprehensive understanding of the As tolerance mechanism using multi-omics analysis. Integrating transcriptomic, proteomic and metabolomics, along with other omics data, we can construct a more comprehensive and systematic regulatory network in response to rice As toxicity, identify the key regulatory nodes and signaling pathways and provide more precise targets and strategies for low-As breeding in rice.

The application of spatial transcriptome analysis. Spatial transcriptome analysis facilitates the resolution of the spatial specificity of gene expression and the identification of the spatial distribution of key gene regulatory networks. Additionally, it allows for an understanding of the effects of As toxicity on plant growth and development in various tissues from a spatial dimension.

The promotion of molecular marker-assisted breeding based on transcriptome information. Information regarding novel genes and/or discovered functional genes is obtained using transcriptome analysis, and highly efficient molecular markers are developed and applied in low-As rice breeding. Superior varieties can be rapidly selected by screening rice materials harboring specific molecular markers, which significantly improves breeding efficiency and accuracy.

The promotion of genome-modification breeding. After identifying As stress-responsive genes using forward genetics methods and/or omics approaches like transcriptome analysis, we verify the availability of these genes via genome-modification technology. Eventually, we aim to reduce the As content in rice grains without sacrificing yields and other agronomic traits (or even increasing yields).

**Figure 3:**
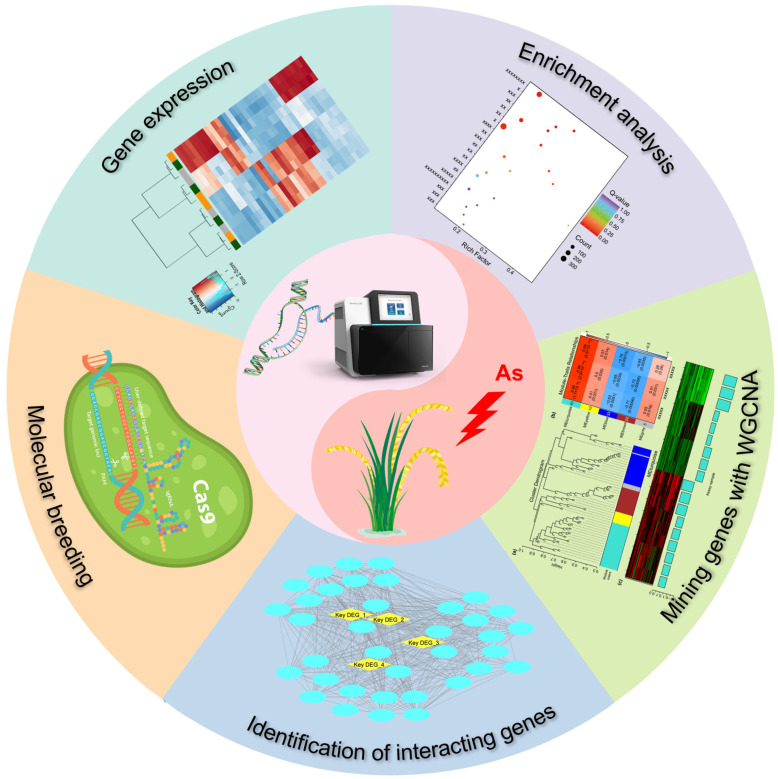
The application of transcriptome analysis in the realm of low-As rice breeding.

In summary, transcriptome analysis has furnished powerful tools and copious data in support of low-As breeding in rice. It has achieved remarkable advances in simultaneously monitoring the expression levels of a group of functional genes, revealing secondary metabolite biosynthesis pathways enriched with DEGs, mining target genes and identifying interacting genes, all of which contribute to molecular breeding (Figure 3). Nevertheless, numerous challenges remain, necessitating continuous efforts in technological innovation, in-depth exploration of mechanisms, bioinformatics analysis and data interpretation. In the future, it is anticipated that the breeding and dissemination of high-yielding and low-arsenic rice varieties will be accelerated by integrating transcriptome analysis and forward genetics methods, applying multi-omics approaches and/or spatial transcriptome analysis and expediting the process of molecular breeding. This will substantially contribute to resolving the issue of rice production in As-contaminated areas and ensuring food security.

## Figures and Tables

**Table 1:** Previously detected QTLs conferring As tolerance in rice.

Population Source	Population Type	Chromosomes	QTL Number	Phenotypes Detected	References
Cheongcheong × Nagdong (120 individuals)	DH	1, 2, 3, 6, 7, 8, 9, 11 and 12	17	SL, RL, CHC, SFW and RFW	[42]
313 accessions from the Hunan Provincial Gene Bank	AMP	1, 4, 5 and 12	5	As accumulation in polished rice	[43]
202 accessions from the USDA-ARS Rice Minicore Collection	AMP	1, 2, 3, 4, 6, 7, 8, 10 and 11	15	As concentration in grains	[44]
307 accessions from the 3K Rice Genomes Project	AMP	All	72	As concentrations in brown and milled rice in normal and selenium treatment conditions, as well as ratio of concentrations under selenium treatment to normal conditions	[45]
273 accessions from the RDA-Genebank	AMP	1, 5 and 6	3	As content in brown rice	[46]
Lemont × TeQing (117/121 individuals)	TIL	4, 5, 8, 9, 11 and 12	7	Inorganic As concentrations in whole-grain brown rice grains	[47]
WTR1 × Hao-an-nong (230 individuals)	BRILs	1, 2, 5, 6, 8 and 9	9	As content in shoots and roots and relative chlorophyll content	[48]
266 accessions from the Bengal and Assam Aus Panel	AMP	All	74	As content in shoots and grains	[49]
276 accessions from a global-wide rice diversity panel	AMP	1, 2, 3, 4, 5, 6, 9, 11 and 12	22	As concentration in grains	[50]
BRRI dhan45 × BRRI dhan47 (1280 individuals)	F_2:3_	8 and 12	5	Average As tolerance indices for shoot length, root length and root–shoot biomass	[51]
Padi Perak × Koshihikari (100 individuals)	F_2_	6 and 8	3	DMA concentrations in rice grains	[52]
Bala × Azucena (105 individuals)	RIL	1, 3, 5 and 6	5	As concentration in grains and leaves	[53]
CJ06 × TN1 (82 individuals)	DH	2, 3, 6 and 8	4	As concentration in shoots, roots and grains	[54]
Bala × Azucena (108 individuals)	RIL	6	1	Maximum root length	[55]

Note: DH, AMP, TIL, BRILs, RIL, SL, RL, CHC, SFW, RFW and DMA are abbreviations for double haploid lines, association mapping population, TeQing-into-Lemont backcross introgression lines, BC_1_F_6_ backcross recombinant inbred lines, recombinant inbred lines, shoot length, root length, chlorophyll content, shoot fresh weight, root fresh weight and dimethyl arsenic acid, respectively.

**Table 2:** Functional genes controlling As uptake, transport and metabolism in rice.

Gene	Accession Number	Chr	Subcellular Location	Manipulation	Effects	References
*OsLsi1*	*Os02g0745100*	2	Plasma membrane	Knockout	Reduced As content in roots and shoots	[56]
*OsLsi2*	*Os03g0107300*	3	Plasma membrane	Knockout	Reduced As content in grains and shoots	[56,57]
*OsNIP1;1*	*Os02g0232900*	2	Plasma membrane	Overexpression	Reduced As content in grains, husks, leaves and shoots	[58]
*OsNIP3;1*	*Os10g0513200*	10	Plasma membrane	Knockout	Reduced As content in various tissues (including grains)	[59]
*OsNIP3;2*	*Os08g0152000*	8	Plasma membrane	Knockout	Reduced As content in roots	[60]
*OsNIP3;3*	*Os08g0152100*	8	Plasma membrane	Overexpression	Reduced As content in grains, husks, leaves and shoots	[58]
*OsPT1*	*Os03g0150600*	3	Plasma membrane	Mutation	Reduced As content in shoots	[61]
*OsPT4*	*Os04g0186400*	4	Plasma membrane	Knockout	Reduced As content in grains and roots	[62]
*OsPT8*	*Os10g0444700*	10	Plasma membrane	Knockout	Reduced As(V) content in roots	[63]
*OsHAC1;1*	*Os02g0102300*	2	Nucleus and plasma membrane	Overexpression	Reduced As content in grains, enhanced As(III) efflux	[64]
*OsHAC1;2*	*Os04g0249600*	4	Nucleus and plasma membrane	Overexpression	Reduced As content in grains, enhanced As(III) efflux	[64]
*OsHAC4*	*Os02g0157600*	2	Nucleus and cytosol	Overexpression	Reduced As content in roots and shoots, enhanced As(III) efflux	[65]
*OsCLT1*	*Os01g0955700*	1	Envelope membrane of plastids	Knockout	Reduced As(III) and As(V) content in roots, increased As(V) content in shoots	[66]
*OASTL-A1*	*Os03g0747800*	3	Cytosol	Knockout	Reduced As content in roots, increased As content in shoots	[67]
*OsASTOL1*	*Os12g0625000*	12	Chloroplast	Knockout	Enhanced sulfur and selenium uptake and reduced As content in grains	[68]
*OsPCS1*	*Os05g0415200*	5	Cytosol	Overexpression	Reduced As content in grains	[69]
*OsPCS2*	*Os06g0102300*	6	Cytosol	Knockdown	Increased As content in shoots	[69]
*OsPRX38*	*Os03g0235000*	3	Apoplast	Heterologous overexpression	Enhanced *Arabidopsis* As tolerance	[70]
*OsGrx_C2.1*	*Os02g0618100*	2	-	Overexpression	Reduced As content in grains	[71]
*OsGrx_C7*	*Os01g0368900*	1	-	Overexpression	Reduced As content in grains	[71,72]
*OsABCC1* (*OsHAG1*)	*Os04g0620000*	4	Tonoplast	Knockout	Increased As content in grains and reduced As content in shoots	[73,74]
*OsABCC7*	*Os04g0588700*	4	Plasma membrane	Knockout	Reduced As content in the aboveground, while no significant change in As content of roots was observed	[75]
*OsPHF1*	*Os07g0187700*	7	Endoplasmic reticulum	Knockout	Enhanced As tolerance	[76]
*OsARM1*	*Os05g0442400*	5	Nucleus	Overexpression	Increased As content in roots	[77]
*OsWRKY28*	*Os06g0649000*	6	Nucleus	Knockout	Reduced As content in shoots	[78]
*OsPIP2;4*	*Os07g0448100*	7	-	Heterologous overexpression	Enhanced *Arabidopsis* As tolerance, while no significant changes in As content of roots and shoots were observed	[79]
*OsPIP2;6*	*Os04g0233400*	4	-	Knockdown	Reduced As content in grains, shoots and flag leaves	[79,80]
*OsPIP2;7*	*Os09g0541000*	9	-	Heterologous overexpression	Enhanced *Arabidopsis* As tolerance, while no significant changes in As content of roots and shoots were observed	[79]
*OsHIR1*	*Os10g0406200*	10	Cell membrane and nucleus	Heterologous overexpression	Reduced As content in *Arabidopsis* roots and shoots	[81]
*OsNLA1*	*Os07g0673200*	7	Cell membrane, cytosol and chloroplast	Knockout	Increased As content in roots and shoots	[82]
*OsNRAMP1*	*Os07g0258400*	7	Cell membrane	Heterologous overexpression	Increased *Arabidopsis* As tolerance and mediated As transfer from roots to shoots	[83]
*OsNPF5.8*	*Os06g0239300*	6	-	Mutation	Enhanced As tolerance by promoting the transport of PC-As(III) complexes into vacuoles	[84]
*OsPTR7* (*OsNPF8.1*)	*Os01g0142800*	1	Cell membrane	Knockout	Reduced As content in grains	[85]
*OsVOZ1*	*Os01g0753000*	1	Nucleus	Knockout	Reduced As content in grains	[86]
*OsVOZ2*	*Os05g0515700*	5	Nucleus and cytosol	Knockout	Reduced As content in grains	[86]
*OsSAUR2*	*Os01g0768333*	1	-	Knockout	Enhanced As uptake in roots and reduced As content in grains	[87]
*SNAC3*	*Os01g0191300*	1	Nucleus	Overexpression	Enhanced As tolerance and increased yields	[88]

Note: As, As(III), As(V) and PC are abbreviations for arsenic, arsenite, arsenate and phytochelatin, respectively.
